# Erythema Nodosum Associated With Streptococcal Infection in Pregnancy

**DOI:** 10.1155/S1064744995000536

**Published:** 1995

**Authors:** W. Edward Richards, Mark B. Reedy, Kevin P. Huddleston, Jeffrey W. Jundt

**Affiliations:** 1 Department of Obstetrics and Gynecology Scott & White Clinic and Memorial Hospital Scott, Sherwood, and Brindley Foundation Texas A&M University Health Science Center College of Medicine Temple TX USA; 2 Department of Internal Medicine Scott & White Clinic and Memorial Hospital Scott, Sherwood, and Brindley Foundation Texas A&M University Health Science Center College of Medicine Temple TX USA

## Abstract

*Background:* Erythema nodosum (EN) is a condition characterized by the presence of painful erythematous nodules on the pretibial aspects of the lower extremities. EN is thought to be a local inflammatory, immune-mediated reaction to a number of systemic antigenic stimuli. This condition is noted most often in women between menarche and menopause and is associated with certain drugs, infections, and pregnancy. However, no reports in the literature describe EN as a result of streptococcal infection during pregnancy.

*Case:* A 21-year-old, white woman, G_3_P_0020_, presented at 13 weeks gestation with a 2-week history of erythematous, tender lesions on the pretibial aspects of both legs consistent with EN. The patient reported having had a “flu-like” illness at the same time the lesions developed. The “flu” symptoms resolved within 10 days without medical intervention, but the lesions on her legs persisted. An initial antistreptolysin-O (ASO) titer was elevated at 960 Todd units (normal values: preschool and adults <85; school-age and young adults <170). Six days later, she presented to the emergency department with complaints consistent with a urinary-tract infection. She was empirically treated with a 10-day course of amoxicillin, 500 mg t.i.d. Although the patient was treated with amoxicillin for a presumed urinary-tract infection (which was culture-negative), the lesions resolved after her completion of the antibiotics. Twelve weeks later, a repeat ASO was within normal limits. The EN lesions did not recur.

*Conclusion:* Although many etiologic factors are identified as causes of EN, the condition is usually self-limiting, requiring only minimal supportive measures until it resolves. A careful history should be obtained and a physical examination performed to exclude other causes. If a recent streptococcal infection is identified or presumed, a 10- to 14-day course of antibiotics is warranted.


Infectious Diseases in Obstetrics and Gynecology 3:166–168 (1995)
© 1995 Wiley-Liss, Inc.

### Erythema Nodosum Associated With Streptococcal Infection in Pregnancy

**W. Edward Richards, Mark B. Reedy, Kevin P. Huddleston, and Jeffrey W. Jundt**

*Departments of Obstetrics and Gynecology (W.E.R., M.B.R., K.P.H.) and Internal Medicine (J.W.J.), Scott & White Clinic and Memorial Hospital, Scott, Sherwood, and Brindley Foundation, Texas A&M University Health Science Center College of Medicine, Temple, TX*

### Abstract

***Background:*** **Erythema nodosum (EN) is a condition characterized by the presence of painful erythematous nodules on the pretibial aspects of the lower extremities. EN is thought to be a local inflammatory, immune-mediated reaction to a number of systemic antigenic stimuli. This condition is noted most often in women between menarche and menopause and is associated with certain drugs, infections, and pregnancy. However, no reports in the literature describe EN as a result of streptococcal infection during pregnancy.**

***Case:*** **A 21-year-old, white woman, $G_3$ $P_{0020}$, presented at 13 weeks gestation with a 2-week history of erythematous, tender lesions on the pretibial aspects of both legs consistent with EN. The patient reported having had a "flu-like" illness at the same time the lesions developed. The "flu" symptoms resolved within 10 days without medical intervention, but the lesions on her legs persisted. An initial antistreptolysin-O (ASO) titer was elevated at 960 Todd units (normal values: preschool and adults <85; school-age and young adults <170). Six days later, she presented to the emergency department with complaints consistent with a urinary-tract infection. She was empirically treated with a 10-day course of amoxicillin, 500 mg t.i.d. Although the patient was treated with amoxicillin for a presumed urinary-tract infection (which was culture-negative), the lesions resolved after her completion of the antibiotics. Twelve weeks later, a repeat ASO was within normal limits. The EN lesions did not recur.**

***Conclusion:*** **Although many etiologic factors are identified as causes of EN, the condition is usually self-limiting, requiring only minimal supportive measures until it resolves. A careful history should be obtained and a physical examination performed to exclude other causes. If a recent streptococcal infection is identified or presumed, a 10- to 14-day course of antibiotics is warranted.**
© 1995 Wiley-Liss, Inc.

Key words
Inflammatory skin disease, panniculitis, erythematous nodules

Erythema nodosum (EN) is an inflammatory disease of the skin and subcutaneous tissue. EN is believed to be an immune-mediated panniculitis that occurs in response to certain disease manifestations such as leprosy, histoplasmosis, tuberculosis, and inflammatory bowel disease; drugs; and normal physiologic processes such as pregnancy.[1] Although EN occurs in men and women, it primarily affects women in their reproductive years. Several reports have suggested a relationship between the occurrence of EN and fluctuations in reproductive hormones, both during the menstrual cycle and in pregnancy.[1–9] An association of EN with oral contraceptives has also been reported.[1–4]

Address correspondence/reprint requests to Publications Office, Scott and White Memorial Hospital, 2401 South 31st Street, Temple, TX 76508.

Received July 10, 1995
Accepted September 7, 1995

One of the most commonly reported causes of EN in adults is streptococcal infection. However, no cases have been reported describing EN associated with a streptococcal infection [positive streptococcal culture or antistreptolysin-O (ASO) titer] during pregnancy.

### CASE

A 21-year-old white woman, $G_3$ $P_{0020}$, was initially evaluated for routine prenatal care at 9 weeks gestation. Her prenatal laboratory tests and cultures were within normal limits.

The patient presented at 13 weeks gestation with the chief complaint of "painful knots on her legs." She stated that 2 weeks earlier she had had a "flu-like" illness consisting of low-grade fever, chills, malaise, rhinitis and sinusitis symptoms, and pharyngitis. She did not seek medical care for this condition, nor did she take antibiotics or over-the-counter medications. Two days after the onset of her "flu" symptoms, she noticed the sudden development of painful pretibial lesions. She had no history of such lesions.

Her medical history was unremarkable. She related no family history of EN and reported no drug allergies. Her obstetrical history was significant for a termination of pregnancy and a spontaneous abortion, each resulting in a D&C at 15 weeks gestation. The patient had used oral contraceptives for several years. No symptoms consistent with EN occurred during her oral-contraceptive use nor during these 2 pregnancies. The father of the index pregnancy was the father of the previous 2 gestations.

On physical examination, the only abnormal findings were several 2–3 cm raised, painful, erythematous nodules on the pretibial aspects of her lower extremities (Fig. 1). All other physical findings were normal.

The laboratory tests included a urinalysis, which was normal. A complete blood cell count showed a WBC count of 8,800/ml with a normal differential and a hemoglobin of 11.9 gm/dl with normal indices. The platelet count was 280,000/ml. An antinuclear antibody titer was negative. The pharyngeal culture was lost, but the ASO titer was 960 Todd units (the reciprocal of the greatest dilution with a detectable antigen). The high ASO titer is pathognomonic for streptococcal infection. The physical findings, history, and laboratory results suggested that her EN could be related to a recent streptococcal infection. Our attempts to notify the patient of our findings were unsuccessful.

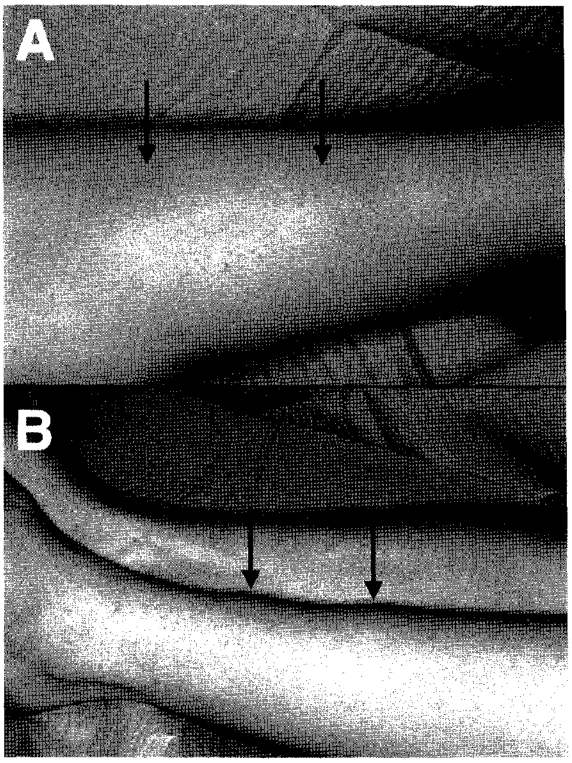

Fig. 1. **A:** Anterior view showing erythematous nodules (arrows). **B:** Lateral view showing nodularity (arrows).

The patient presented again to the emergency department 6 days after the diagnosis of EN with symptoms of a urinary-tract infection. The EN lesions were still present and tender. A urine culture was obtained, and she was treated empirically with amoxicillin, 500 mg t.i.d. for 10 days, for a presumed urinary-tract infection. Three days later, she was notified that the urine culture was negative. At that time, the patient reported a marked improvement in her EN lesions (less painful and smaller). She was instructed to complete the full antibiotic course. The lesions had completely resolved upon her completion of the antibiotics and did not recur during the remainder of her pregnancy. A repeat ASO titer 12 weeks later was normal. The patient subsequently had an uncomplicated spontaneous vaginal delivery at 41 weeks gestation. Both she and her infant were discharged home on postpartum day 2.

### DISCUSSION

EN is the most common form of nodular panniculitis.[6,7] Clinically, a patient with EN presents with a sudden onset of raised, tender, erythematous nodules on the pretibial aspects of the lower extremities. The nodules vary in size from 2 to 10 cm. The normal panniculus, which is located in the space below the dermis and above the fascia, is composed of adipose tissue in lobules. Each lobule has a central artery. A fibrous septum containing blood vessels, nerves, and lymphatics surrounds the adipose lobule and the central artery complex. The histologic and pathologic features of EN show an initial perivascular septal inflammatory infiltrate of neutrophils.[6,7] Later, these neutrophils are replaced by lymphocytes, ultimately becoming a granulomatous infiltrate as the lesion ages. Typically, EN is a self-limiting condition that resolves spontaneously within 6 weeks.

EN results from an immune response to a variety of antigenic stimuli.[1,8,9] Our patient presented an interesting case because several possible stimuli were present. One stimulus was the pregnancy itself, since hormone fluctuations in pregnancy may be linked to the development of EN.[1–4] However, pregnancy was not believed to be the stimulus because of her 2 prior 15-week gestations with the same father in which EN did not occur. Furthermore, the entire EN course during the index pregnancy occurred before 15 weeks gestation.

Oral contraceptives have also been implicated as a stimulus for EN.[1–3] This patient had taken oral contraceptives intermittently for 7 years prior to the conception of the index pregnancy, but at no time during those 7 years did she develop EN. Moreover, oral contraceptives were not believed to have been responsible for this episode since EN did not develop until at least 3 months after her last exposure to oral contraceptives.

When our patient presented with painful lesions of the lower extremities and a history of a recent upper respiratory infection, we felt that the antigenic stimulus for EN could have been a streptococcal infection. The patient's elevated ASO titer provided further support for a recent streptococcal infection. During her treatment for a suspected urinary-tract infection, the EN symptoms markedly diminished and completely resolved after her completion of a 10-day antibiotic course. Twelve weeks later, her repeat ASO titer was normal.

When EN is observed in pregnancy, a careful history and physical examination must be completed to address each possible stimulus. If EN is felt to be associated with a streptococcal infection (positive culture or ASO titer), a 10- to 14-day course of antibiotics is warranted. Other possible causes should be evaluated and treated as appropriate in the obstetrical patient.

### REFERENCES

1. Bartelsmeyer JA, Petrie RH: Erythema nodosum, estrogens, and pregnancy. Clin Obstet Gynecol 33:777–781, 1990.
2. Bombardieri S, Munno OD, Di Punzio C, Pasero G: Erythema nodosum associated with pregnancy and oral contraceptives. Br Med J 1:1509–1510, 1977.
3. Salvatore MA, Lynch PJ: Erythema nodosum, estrogens, and pregnancy. Arch Dermatol 116:557–558, 1980.
4. Langer R, Bukovsky I, Lipshitz I, Ariely S, Caspi E: Erythema nodosum associated with pregnancy. Case reports. Eur J Obstet Gynaecol Reprod Biol 9:399–401, 1979.
5. Blomgren SE: Conditions associated with erythema nodosum. J Med 72:2302–2304, 1972.
6. Soter NA, Franks AG Jr: The skin and rheumatic diseases. In Kelley WN, Harris ED, Ruddy S, Sledge CB (eds): Textbook of Rheumatology. 4th Ed. Philadelphia: W.B. Saunders Co., pp 529–530, 1993.
7. Hoffman BI: Panniculitis and related disorders of subcutaneous fat. In Katz WA (ed): Diagnosis and Management of Rheumatic Diseases. 2nd Ed. Philadelphia: J.B. Lippincott Co., pp 735–736, 1988.
8. el-Zawahry M: Erythema nodosum: A study of 60 cases. Int J Dermatol 10:145–150, 1971.
9. Jillson OF: Erythema nodosum. J Maine Med Assoc 64: 264–265, 1973.
